# Complications of invasive mechanical ventilation in critically Ill Covid-19 patients - A narrative review

**DOI:** 10.1016/j.amsu.2022.104201

**Published:** 2022-07-16

**Authors:** Wajiha Khan, Adnan Safi, Muhammad Muneeb, Mehwish Mooghal, Ali Aftab, Jawad Ahmed

**Affiliations:** a Department of Medicine and Surgery, Dow University of Health and Sciences, Baba-e-Urdu Road, Karachi, 74200, Pakistan; b Department of Medicine and Surgery, Nishtar Medical University and Hospital, Nishtar Road, 60000, Multan, Pakistan; c Department of Surgery, PNS Shifa Hospital. DHA Phase 2, Sailors Street, Karachi, 75500, Pakistan; d Department of Surgery, Bahria University Medical and Dental College. DHA Phase 2, Sailors Street, Karachi, 75500, Pakistan; e Department of Internal Medicine, Dow University of Health Sciences, Baba-e-Urdu Road, Karachi, 74200, Pakistan

**Keywords:** Covid-19, Coronavirus disease, Critically ill, Intensive care unit, Invasive mechanical ventilation, IMV, Invasive Mechanical Ventilation, LPV, Lung Protective Ventilation, FTTL, Full Thickness Tracheal Lesions, TEF, Tracheoesophageal fistula, SAPS 2, Simplified Acute Physiology Score 2, SOFA, Sequential Organ Failure Assessment score, VAP, Ventilator Associated Pneumonia

## Abstract

Critically ill COVID-19 patients have to undergo positive pressure ventilation, a non-physiological and invasive intervention that can be lifesaving in severe ARDS. Similar to any other intervention, it has its pros and cons. Despite following Lung Protective Ventilation (LPV), some of the complications are frequently reported in these critically ill patients and significantly impact overall mortality. The complications related to invasive mechanical ventilation (IMV) in critically ill COVID-19 patients can be broadly divided into pulmonary and non-pulmonary. Among pulmonary complications, the most frequent is ventilator-associated pneumonia. Others are barotrauma, including subcutaneous emphysema, pneumomediastinum, pneumothorax, bullous lesions, cardiopulmonary effects of right ventricular dysfunction, and pulmonary complications mimicking cardiac failure, including pulmonary edema. Tracheal complications, including full-thickness tracheal lesions (FTTLs) and tracheoesophageal fistulas (TEFs) are serious but rare complications. Non-Pulmonary complications include neurological, nephrological, ocular, and oral complications.

## Highlights

• The complications related to IMV in critically ill covid 19 patients can be broadly divided into pulmonary and non-pulmonary complications.
• Among pulmonary complications the most frequent is Ventilator associated pneumonia.
• Others are Barotrauma, Cardiopulmonary effects of right ventricular dysfunction & Pulmonary complications mimicking cardiac failure including pulmonary edema, tracheal complications including full thickness tracheal lesions & tracheoesophageal fistulas.
• Non-Pulmonary complications of prolonged IMV include neurological, nephrological, ocular and oral complications.

## 1 Introduction

SARS-CoV-2, the main culprit behind the recent global pandemic, has infected and killed millions of people since the day it was first detected in Wuhan, China in late December 2019. Although most patients infected by this virus encounter only mild symptoms like fever, cough, body aches but Acute Respiratory Distress Syndrome (ARDS) is also not uncommon with this virus.^cc^ [1]. In such cases, Intensive Care Unit (ICU) admission and mechanical ventilation become almost inevitable. About 80% of people suffering from COVID-19 related ARDS required mechanical ventilation at some point during their hospital stay [2]

Mechanical ventilation has been recognized as one of the main lifesaving management options in the recent pandemic. Still, at the same time, this fact cannot be neglected that the mortality rate of mechanically ventilated patients with COVID-19 ranges from 30% to 97% despite using Lung Protective Ventilation in most centers [3,4]. This high mortality rate can partially be explained by multiorgan failure and other complications related to COVID-19, and partially by the complications related to mechanical ventilation itself. Though most of the complications related to Invasive Mechanical Ventilation (IMV) are the same in non-COVID and COVID-19 related mechanically ventilated patients. Some of these complications are seen more frequently, with increased severity and worsened outcomes for patients with COVID-19, ultimately resulting in prolonged ICU stay and increased mortality.

The is review aims to comprehensively discuss the published literature on the complications of IMV in critically ill COVID-19 patients and compare the incidence of these complications with the Non-COVID-19 mechanically ventilated patients if mentioned in the literature.

**Fig. 1:**
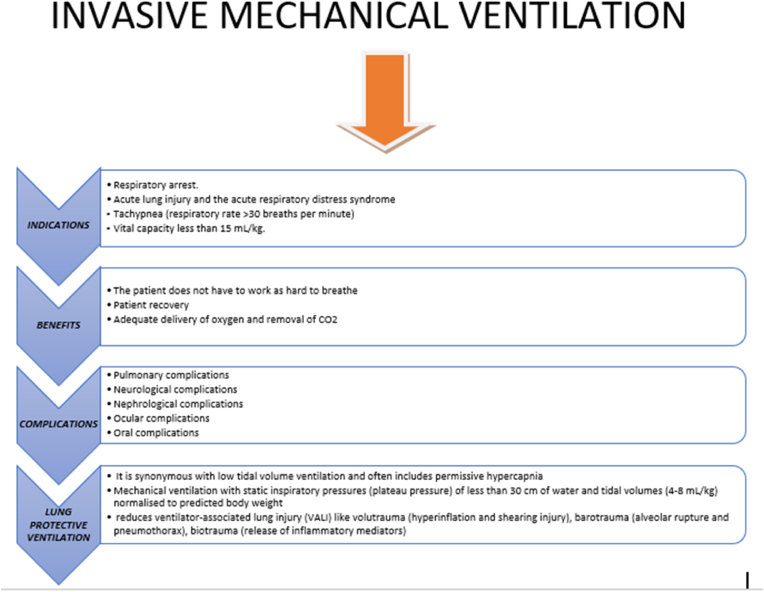
Demonstrates indications, benefits, complications and Lung Protective Ventilation.

The indications, benefits, complications and the concept of Lung Protective Ventilation is shown in Fig. 1.

## 2 Patients’ characteristics for ventilatory support

In a large multicenter, prospective cohort study conducted in France, Belgium, and Switzerland, including data from 149 ICUs, and consisting of a total of 4,244 patients, showed that about 80% of patients admitted in ICU received IMV at some point during their ICU stay (most of the patient had Acute Respiratory Failure). Among these patients, the demographic characteristics found to be significant (p < 0.05) were age, gender, BMI (≥30 kg/m2), comorbidities, immune status, SAPS 2 score, and SOFA score at the time of ICU admission. In the patients who received ventilatory support, the median age was 63 years with the SAPS 2 and SOFA score at the time of ICU admission of 37 and 5, respectively. The most frequent comorbidities that had significant impact were hypertension, known diabetes and immunocompromised status [5]. Homogenous characteristics were recorded by Josefina et al., Ines Gragueb et al. and other researchers. [[6], [7], [8], [9], [10]] Patients’ characteristics observed in different studies are summarized in Table 1.

**Table 1:** Patients' characteristics for ICU admission and ventilatory support.

**Study name**	**Location**	**Total no of patients**	**Mean age (SD) years**	**Males (%)**	**Characteristics of intubated patients**
**COVID-ICU Group on behalf of the REVA Network**	France, Belgium, Switzerland	4,244	63	74.1%	• BMI≥30 kg/m^2^ • Mean SAPS 2 score = 37 • Mean SOFA score = 5 • Comorbids = hypertension (48%), diabetes(28%), immunocompromised (7%) • Smokers (5%)
**Gragueb-Chatti I et al.**	France	151	64	79%	• Mean SAPS 2 score = 42 • Mean SOFA score = 6 • Comorbids = hypertension (48%), diabetes (36%), obesity (29%) • Smokers (25%)
**Gamberini L et al.**	Italy	391	66	77%	• Median BMI = 28kg/m2 • Mean SAPS 2 score = 38 • Mean SOFA score = 5 • Comorbids = hypertension (57%), diabetes(21.7%) • Smokers = 54.4%
**Udi J et al.**	Germany	20	61	94%	–
**Smet J et al.**	Brussels, Belgium	220	53	62%	• Median BMI = 28.1 kg/m2 • Comorbids = hypertension(35%), diabetes(18%) • Smokers (22%)

## 3 IMV settings for Covid-19 patients

ARDS is the main indication of receiving IMV in COVID-19 patients admitted in ICU ^2,5^ARDS seen in COVID-19 patients is no different than the non-COVID-19 patients, though a study suggested that duration of IMV is longer than those with ARDS unrelated to COVID-19 [5,11]. The conception of Lung-protective mechanical ventilation, is to prevent injury from overdistention by using lower tidal volumes and lower inspiratory pressures or injury from ventilation with atelectasis at end-expiration, is also pertinent in the case of ARDS due to COVID-19 and has been successfully practiced by hospitals around the globe. The IMV settings advocated by available data in the first 24 h of ICU admission in COVID-19 patients are: Tidal Volume (V_T_) of 4–8 ml/kg of Predicted Body Weight (PBW), Plateau Pressure of <28 cmH_2_O, FiO2, and PEEP titrated to achieve SpO2 88–92% and static respiratory system compliance (C_RS_) of 31–47 ml/cm H_2_O, and Respiratory Rate of 15–32/min to aim for PaCO_2_ 35–45 mmHg [5,6,8,12,13].

## 4 Complications of IMV in Covid-19 patients

IMV is a breakthrough invention in medicine and has been a glimmer of hope for severely compromised ICU patients during the COVID-19 pandemic. But this lifesaving device simultaneously is associated with severe pulmonary and non-pulmonary complications. Emergency Room doctors and nurses need to know these complications as many of these can be avoided with proper care and management.

A **Pulmonary Complications:**

Pulmonary complications are not of uncommon occurrence in COVID-19 patients who undergo IMV. The incidence of different pulmonary complications is shown in Table 2. Some of the important complications are described below

a **Ventilator-Associated Pneumonia:**

**Table 2:** Incidence of common pulmonary complication in COVID-19 patients undergoing IMV.

**Study name**	**Location**	**Total no of patients included in the study**	**Total no and % of patients with the complication**
**Ventilator Associated Pneumonia (VAP)**			
**Gamberini L et al.**	Italy	391	• Early onset VAP = 76(19.4%) • Late onset VAP = 175(44.8%)
**Gragueb-Chatti I et al.**	France	151	• 91 patients(60%)
**COVID-ICU Group on behalf of the REVA Network**	France Belgium Switzerland	4,244	• 1209 patients (58%)
**Rouzé A et al.**	France Spain Greece Portugal Ireland	1576 patients out of which 568 infected by SARS-CoV-2 and 482 had influenza while 526 = no viral infection	• 287 (50.5%) in patients infected with SARS-Cov-2
**Barotrauma**			
**Udi J et al.**	Germany	20	8 patients(40%) • Pneumothorax(25%) • Pneumomediastinum(25%) • Pneumopericardium(5%) • Extended ssubcutaneous eemphysema (25%)
**Yang X et al.**	Wuhan, China	52	1 patient(5%) • Pneumothorax(5%)
**Wang XH et al.**	China	248	5 patients(2.01%) •Pneumothorax(2.01%)
**Jones E et al.**	London, UK	83	8 patients (9.6%) • Subcutaneous Emphysema(9.6%) • Pneumomediastinum(8.4) • Pneumothorax(4.8%) • Bilateral Pneumothoraces(2.4%)
**McGuinness G et al.**	New York, USA	601	89 patients (15%) • Pneumothorax(9%) • Pneumomediastinum(10%) • Pneumopericadium (2%)
**Cardiopulmonary Effects**			
**Sergio Caravita et al.**	Italy	42	21 Patients (50) • Raised Pulmonary arterial Pressure 16 (76%) • Pulmonary Hypertension 12 (57%)
**Pulmonary complications mimicking Cardiac Failure**			
**Caroline Bleakley et al.**	UK	90	Right Ventricular Dysfunction • Low Right ventricle fractional area change (RV FAC) 75 (72%) • High Right Ventricle systolic pressure (RVSP) 65 (72%)

Among pulmonary complications, Ventilator-Associated Pneumonia (VAP), especially late-onset, has been the most frequent complication of IMV in COVID-19 patients (40–60%) [5,7,8]. Studies even highlighted the higher incidence of VAP in ARDS due to COVID-19 than in “classical ARDS” associated with other pulmonary infections, as with influenza [7,8,14]. This can be explained by more frequent use of immunomodulatory agents or SARS-CoV-2 infection by itself [15,16]. Other reasons can be micro-aspiration from the oropharyngeal cavity and diminished host defense due to decreased cough efficiency and impaired mucociliary clearance due to excessive sedation and prolonged ventilation [17]. Lorenzo et al. even pointed out through their study that VAP itself is associated with lengthening of IMV duration in critically ill covid-19 patients [8]. Sampling and culturing done for VAP associated with COVID-19 is same as that for non- COVID-19 patients, i.e. quantitative distal bronchoalveolar lavage cultures growing≥104 cfu/mL, blind protected specimen brush distal growing≥103 cfu/mL, or endotracheal aspirates growing≥106 cfu/mL before initiation of antibiotics [5]. Microbiological details identified Gram-negative bacteria (66%), especially Enterobacteriaceae and non-fermenting Gram-negative bacilli, as the most common pathogen. Gram-positive pathogens were mainly methicillin-susceptible *Staphylococcus aureus* (MSSA) and Enterococcus spp [7].

b **Barotrauma (Subcutaneous Emphysema, Pneumomediastinum, Pneumothorax, Bullous Lesions):**

Barotrauma, a condition in which alveolar rupture leads to subsequent air entry into surrounding extra-alveolar space, is a term collectively used for subcutaneous emphysema, pneumomediastinum, pneumothorax, bullous lesions, etc. The high incidence of barotrauma in COVID-19 patients compared to non- COVID-19 is a disputable matter as some studies highlighted its increased incidence while others supported the opposite [6,[18], [19], [20], [21]]. Positive Pressure Ventilation (PPV) especially invasive, has grave importance in managing COVID-19 patients with hypoxic respiratory failure leading to severe ARDS. Barotrauma is a known complication of PPV. It may occur due to increased intra-alveolar pressure, high tidal volume, or high intrinsic positive end-expiratory pressure (PEEP) [22]. An alternative pathology in the development of barotrauma has also been suggested in COVID 19 patients with ARDS as most of the patients who developed barotrauma were on lung-protective ventilation, and few of the patients even developed this without any previous exposure to IMV [19,23]. It has been hypothesized that extensive lung damage secondary to an inflammatory response in COVID-19 patients can lead to increase respiratory drive with persistent strong spontaneous inspiratory efforts causing self-inflicted lung injury. When triggered by PPV, it can lead to barotrauma [24]. That is why barotrauma associated with Mechanical Ventilation parameters has been a controversial subject. Many studies found no correlation between the two, while some investigators believe that high PEEP in IMV is associated with increased incidence of this complication [21,25,26]. Whatever the pathology behind this complication is, barotrauma nonetheless is associated with worse clinical outcomes, multiple organ failure, lower discharge rates, and death in COVID-19 patients [20,27].

c **Cardiopulmonary effects:**

Sergio et al. in a retrospective study conducted on twenty-one mechanically ventilated patients who underwent right heart catheterization pointed out that these patients had increased Right Atrial Pressure, with a high ratio between Right Atrial Pressure and Pulmonary Artery Wedge Pressure, as compared to control group (who underwent an elective right heart catheterization for unexplained dyspnoea after a comprehensive non-invasive evaluation) presaging right heart failure [10]. This can be possibly explained by enhanced ventricular interdependence in mechanically ventilated patients because of reduce lung compliance and high PEEP [28].

d **Pulmonary complications mimicking Cardiac Failure:**

\Pulmonary manifestation of Acute Respiratory Distress Syndrome in COVID-19 patients undergoing IMV can also result in cardiac complications including myocarditis, taksotsubo cardiomyopathy, arrhythmias, or acute coronary syndromes. These complications of COVID-19 are thought to be a combination of direct viral injury and the host's immune response resulting in vascular inflammation, plaque instability, and myocardial inflammation [29]. Critically ill COVID-19 patients require spontaneous and mechanical ventilation, which introduces significant modifications in the physiology of heart-lung interactions and induces changes in intrapleural or intrathoracic pressure and lung volume, which can independently affect the key determinants of cardiovascular performance: atrial filling or preload; the impedance to ventricular emptying or afterload; heart rate and myocardial contractility [30]. Spontaneous inspiration produces a negative pleural pressure, and the reduction in intrathoracic pressure is transmitted to the right atrium. In contrast, intermittent positive pressure ventilation (IPPV) produces inspiratory increases in intrathoracic pressure and, therefore right atrial pressure (P_RA_). If a positive end-expiratory pressure (PEEP) is added, these pressures remain greater than atmospheric pressure throughout the respiratory cycle [30]. The subsequent increase in pleural pressure (intrathoracic pressure) exerts opposite actions on the loads of both ventricles, reducing RV preload while increasing LV preload [31]. Pulmonary complication mimicking cardiac failure results in pulmonary edema and right ventricular dysfunction. These known complications are prevalent in the setting of COVID-19 ARDS. Echocardiographic studies have shown that RV dysfunction in Covid-19 takes the form of a specific radial dysfunction [32], and that it is commonly accompanied by RV dilation due to pressure overload. RV dilation occurs in up to 49% of patients, while RV systolic dysfunction occurs in up to 40%. A particular form of radial RV dysfunction may even be observed in up to 70% of patients [32]. There is a big gap of understanding the exact mechanisms resulting in cardiovascular complications in critically ill COVID-19 patients undergoing mechanical ventilation.

e **Tracheal complications due to MV:**

Tracheal complications, including full-thickness tracheal lesions (FTTLs) and tracheoesophageal fistulas (TEFs), are serious but rare complications of prolonged invasive mechanical ventilation [33]. Critically ill COVID-19 patients requiring prolonged mechanical ventilation showed higher incidence of tracheal complication than Non-COVID-19 patients [34]. The FTTLs and TEFs are detected either directly with a bronchoscopy before performing a tracheostomy, or clinically and/or with CT scans in case of onset of other complications, such as pneumothorax, pneumomediastinum, or subcutaneous emphysema [35]. Several mechanisms specifically may explain the greater incidence of tracheal complications in mechanically ventilated patients with COVID-19: a) Early implementation of pronation maneuvers, which increase the cuff pressure on the tracheal walls, b) Prothrombotic and antifibrinolytic state of patients with COVID-19, which may cause microvascular injury and necrosis of tracheal and esophageal mucosa, c) High viral replication within the tracheal epithelium that weakens the mucosa, d) High dose of systemic steroids and their chronic use that may cause mucosal atrophy and alter normal healing of tracheal wall micro-wounds caused by intubation, cuff pressure, or tracheostomy [36], e) The hypoxic damage to the tracheal mucosa witnessed by a lower PaO2/FIO2 ratio in the second week of invasive MV compared with the control group. ^35^However, the exact mechanisms and long-term outcomes need to be further investigated. Following recommendations should be considered in patients with MV patients with Covid-19. First, a bronchoscopy is indicated periodically (eg, weekly) to detect any early signs of tracheal and endobronchial lesions e.g mucosal hyperemia, mucosal ischemia, or ulcer [34]. Second, high steroid doses (intravenous methylprednisolone, 80 mg) should be used with caution, and the cuff pressure should be monitored to avoid hypoperfusion and pressure sores of the tracheal mucosa, particularly when a nasogastric tube is positioned. Third, an adequate clinical and radiological follow-up should be performed in patients treated with prolonged invasive MV to allow early identification [35].

B **Non- Pulmonary Complications (**Table 3**)**

**Table 3:** Incidence of Common Non Pulmonary Complications in COVID-19 patients undergoing IMV.

**Study name**	**Location**	**Total no of patients included in the study**	**Total no and % of patients with the complication**
**Neurological Complications**			
**Battaglini D et al.**	Italy	94	•Delirium 34 (3.17%) •Critical Illness Neuropathy 5 (5.32%) •Coma 4 (4.25%) •Acute Ischemic Stroke 3 (3.25%) •Stupor 3 (3.19) •Seizures 2 (2.13%) •Encephalopathy 2 (2.13%)
**Ely EW et al.**	USA	224	•Delirium 183 (81.7%) •Coma 24 (10.71%)
**Nephrological Complications**			
**Sang L et al.**	China	210	•AKI 92 (41.07%)
**Lombardi R et al.**	Uruguay	2783	•AKI 803 (28.85%) •ARF 253 (9.09%)
**Oral Complications**			
**Sleiwah A et al.**	UK	16	•Peri Oral Ulcer 16 (100%)

a) **Neurological Complications:**

COVID-19 primarily affects respiratory system, including dry cough, fever, fatigue, and respiratory failure. However, recent data suggest that COVID-19 is not confined to the airways but is also responsible for possible neurological involvement in patients undergoing prolonged invasive mechanical ventilation [[37],^38^]. Virus may pass to the central nervous system by different routes, including hematogenous spread from the systemic to the cerebral circulation and lymphocyte invasion or dissemination from the cribriform plate and olfactory bulb to the brain [39]. It seems to be consistent with the loss of smell and taste, quite prevalent—presentations of COVID-19 [40]^..^ A recent systematic review of 37 articles revealed that 20% of COVID-19 patients undergoing IMV present with headache, 60% with anosmia/ageusia, 25% with myalgia/myositis, 8.8% with encephalopathy, 2.8% with ischemic stroke, and 0.45% with intracerebral hemorrhage, other neurological symptoms include impaired consciousness, ataxia, seizures, and neuralgia [43]. In another cohort study, neurological complications were detected in half of the patients admitted to ICU with confirmed COVID-19 pneumonia who undergoes mechanical ventilation. The most frequent complication was delirium (36.70%), followed by coma, critical illness neuropathy, ischemic stroke, stupor, encephalopathy, seizures, cognitive deficit, and depression [44]. Delirium is known to be associated with longer ICU stay and mechanical ventilation days and an increased risk of death at six months, disability, and long-term cognitive dysfunction [45]. The use of sedating medications in critically ill patients, especially sedative-hypnotics and anticholinergic agents, is associated with the development of delirium [46]. Transcranial Doppler (TCD) ultrasonography, Optic Nerve Sheath Diameter (ONSD) measurement, and quantitative automated pupillometry are safe, useful methods that can be applied at the patient's bedside to assess cerebral hemodynamics as well as to monitor cerebral perfusion pressure and intracranial pressure noninvasively [41,42].

b) **Nephrological complications:**

Various nephrological complications occur in critically ill patients, among which Acute Kidney Injury (AKI) is a frequent complication in COVID-19 patients. AKI reportedly correlated with poor clinical outcomes in patients with COVID-19 compared to Non-COVID-19 patients. It affects up to 29% of patients who are mechanically ventilated [49]. Factors involved are age, the presence of sepsis, use of nephrotoxic drug, IMV, and baseline serum creatinine levels were associated with the development of AKI. Recently, the association between IMV and AKI in ARDS patients has become widely acceptable. Several studies had postulated that IMV might have played a key role in the development of AKI due to the mechanisms of lung-kidney cross-talk [50]. Hypercapnia is common in patients with AKI undergoing IMV; It is challenging to correct for the hypercapnia even if the patients had been intubated and received lung protective ventilation strategy by adjusting the tidal volume and plateau pressure. This might explain why IMV is the strongest risk factors associated with AKI [51].

c) **Ocular Complications**

Ocular complications in mechanically ventilated COVID-19 patients mostly include disorders involving the eye's surface, i.e., cornea. The cornea is the main refractive power of the eye, and its disorders can ultimately lead to blindness or a decrease in vision. Some of the most prevalent disorders include exposure keratopathy, conjunctivitis, and keratitis [52].

Exposure keratopathy is corneal damage due to prolonged exposure to the external environment. Patients who are under mechanical ventilation are heavily sedated, decreasing their ability to properly close the eyelid (lagophthalmos), exposing their ocular surface, and leading to exposure keratopathy [52].

Infective conditions like conjunctivitis and keratitis in mechanically ventilated patients occur due to colonization of the eye with bacteria and secretion from the respiratory tract. This colonization usually occurs during the process of endotracheal suctioning. The most common organism involved is *Pseudomonas aeruginosa*, followed by Staphylococcus epidermidis and Acinetobacter spp [52].

d) **Oral Complications**

One of the complications observed in COVID-19 patients requiring long-term mechanical ventilation is the involvement of oropharyngeal mucosa. Hemorrhagic ulcers, perioral pressure ulcers, and macroglossia are the most reported complaints [53].

According to a study conducted in Slovakia, 3 out of 9 COVID patients admitted in the ICU were found to have hemorrhagic ulcers in their oral cavities and in their perioral regions. The most common organism responsible for these hemorrhagic ulcers was *Pseudomonas aeruginosa*, followed by *Klebsiella pneumoniae* and *Enterococcus faecalis*. These ulcers mostly developed on the dorsal surface of the tongue [53].

Pressure ulcers are injuries to the skin caused by prolonged pressure. A perioral pressure ulcer is the most prevalent oral complication associated with mechanical ventilation in COVID-19 patients [[53], [54], [55]]. According to a study published in European Journal of Plastic Surgery, these ulcers not only developed due to the mechanical ventilation itself but also due to the devices used to secure the endotracheal tubes [56].

Macroglossia is an abnormal enlargement of the tongue. Long-term invasive ventilation in COVID-19 patients causes lingual compression and edema leading to macroglossia. However, according to a case report published in BMJ, the most important risk factor for macroglossia in Covid patients is prolonged pronation cycles [57].

## 5 Conclusion

COVID-19 pandemic is the ongoing public health crisis. Though most patients present with mild symptoms, ARDS is also not unusual with SARS-CoV-2 infection. In case of severe ARDS, the application of IMV becomes inevitable. The majority of the ICUs around the globe are exercising the concept of Lung Protective Ventilation to minimize the chances of Ventilator-associated complications, which have a negative impact on the disease course and mortality. However, ventilator-associated complications are still frequently reported in critically ill COVID 19 patients. Highlighting these complications is imperative so that proper measures can be taken to address these complications as this will drop down the mortality rate associated with COVID-19 significantly.

## Ethical approval

Not applicable.

## Sources of funding

This research has not been sponsored and funded by any group or a person.

## Author contribution

Wajiha Khan: Contribution to the concept/design; acquisition, analysis, and interpretation of data, drafting and revision of article for the content and final approval before submission and accountable for all aspects of work. Adnan Safi: Contribution to the concept/design; acquisition, analysis, and interpretation of data, drafting and revision of article for the content and final approval before submission. Muhammad Muneeb: Contribution to the concept/design; acquisition, analysis, and interpretation of data, drafting and revision of article for the content. Mehwish Mooghal: Contribution to the concept/design; acquisition, analysis, and interpretation of data, drafting and revision of article for the content. Ali Aftab: Contribution to acquisition, analysis, and interpretation of data, drafting and revision of article for the content. Jawad Ahmed: Contribution to acquisition, analysis, and interpretation of data, drafting and revision of article for the content.

## Consent

Not applicable.

## Trail registry number

1. Name of the registry:

2. Unique Identifying number or registration ID:

3. Hyperlink to your specific registration (must be publicly accessible and will be checked):

## Guarantor

Dr. Wajiha Khan and Dr. Mehwish Mooghal.

## Consent for publication

Not applicable.

## Provenance and peer review

Not commissioned, externally peer reviewed.

## Research Questions

• What are the patients' characteristics associated with ICU admission and IMV in SARS-CoV-2 infection?
• What are the most frequent pulmonary complications reported in COVID-19 patients on IMV?
• What are the non-pulmonary complications seen in COVID -19 patients on IMV?

## Declaration of competing interest

We do not have any conflicts of interest.
